# Personalizing first-line treatment in advanced colorectal cancer: Present status and future perspectives

**Published:** 2021-11-29

**Authors:** Rodrigo Motta, Santiago Cabezas-Camarero, Cesar Torres-Mattos, Alejandro Riquelme, Ana Calle, Paola Montenegro, Miguel J. Sotelo

**Affiliations:** ^1^Department of Medical Oncology, Aliada Cancer Center, Lima, Peru; ^2^Instituto Nacional de Enfermedades Neoplasicas, Lima, Peru; ^3^Department of Medical Oncology, Hospital Universitario Clínico San Carlos, Instituto de Investigación Sanitaria San Carlos, Madrid, Spain; ^4^Department of Medical Oncology, Hospital Nacional Guillermo Almenara Irigoyen, Lima, Peru; ^5^Oncological Research Unit, Clínica San Gabriel, Lima, Peru; ^6^Department of Medical Oncology, Hospital Universitario Infanta Cristina, Madrid, Spain; ^7^Department of Medical Oncology, Hospital María Auxiliadora, Lima, Peru; ^8^Auna-OncoSalud Network, Lima, Peru

**Keywords:** metastatic colorectal cancer, personalized therapy, precision medicine, first-line, mutation

## Abstract

**Background:**

Colorectal cancer is one of the most frequent neoplasms worldwide, and the majority of patients are diagnosed in advanced stages. Metastatic colorectal cancer (mCRC) harbors several mutations with different prognostic and predictive values; *KRAS*, *NRAS*, and *BRAF* mutations are the best known. Indeed, *RAS* and *BRAF* molecular status are associated with a different response to monoclonal antibodies (Anti-epidermal growth factor receptor and anti-vascular endothelial growth factor receptor agents), which are usually added to chemotherapy in first-line, and thus allow to select the optimal therapy for patients with mCRC. Furthermore, sidedness is an important predictive and prognostic factor in mCRC, which is explained by the different molecular profile of left and right-sided tumors. Recently, microsatellite instability-high has emerged as a predictive factor of response and survival from immune checkpoint inhibitors in mCRC. Finally, several other alterations have been described in lower frequencies, such as human epidermal growth factor receptor-2 overexpression/amplification, *PIK3CA* pathway alterations, phosphatase and tension homolog loss, and hepatocyte growth factor/mesenchymal-epithelial transition factor pathway dysregulation, with several targeted therapies already demonstrating activity or being tested in currently ongoing clinical trials.

**Aim:**

To review the importance of studying the predictive and prognostic roles of the molecular profile of mCRC, the changes occurred in recent years and how they would potentially change in the near future, to guide physicians in treatment decisions.

**Relevance for Patients:**

Today, several different therapeutic options can be offered to patients in the first-line setting of mCRC. Therapies at present approved or under investigation in clinical trials will be thoroughly reviewed, with special emphasis on the molecular rationale behind them. Understanding the molecular status, resistance mechanisms and potential new druggable targets may allow physicians to choose the best therapeutic option in the first-line mCRC.

## 1. Introduction

In 2020, there were 1,931,590 new cases of colorectal cancer (CRC), accounting for 10% of all new cases of cancer worldwide, with the third and second most frequent incidences in men and women, respectively. Furthermore, CRC caused 935,173 deaths in 2020, making it the malignant neoplasm with the fifth highest mortality worldwide. This high mortality rate is explained because a majority of cases are diagnosed in an advanced stage [1].

CRC develops more frequently in patients over 50 years of age, especially in those with a history of smoking, alcoholism, obesity, heavy red meat consumption, and lack of physical activity [2]. Although its exact origin is not yet known, CRC develops in the context of well-known acquired genetic aberrations, some of which have been shown to be prognostic while some others allow to predict the benefit from different biological agents [3,4].

*RAS* and *BRAF* mutational status and the analysis of microsatellite instability (MSI) are mandatory in patients with metastatic CRC (mCRC) for prognostic as well as therapeutic purposes. Moreover, some other genetic alterations are increasingly being tested to expand the array of druggable alterations in current daily practice in mCRC, and several agents against some other potentially targetable genetic aberrations are being tested in clinical trials [5,6]. Applying one of Sun Tzu’s principles from his *Art of War* (“Know your enemy and know yourself, and you will be victorious in a thousand battles”), we aim to dissect the molecular biology and druggable mutational landscape of CRC to guide treatment decisions in the first-line setting, as well as its future perspectives.

## 2. Knowing Your Enemy: Molecular Pathways and Mutational Status in mCRC

The past 15 years saw the advent of the biological therapies for mCRC through the appearance of anti-vascular endothelial growth factor (VEGF) and anti-epidermal growth factor receptor (EGFR) agents. However, it was soon evidenced that not all mCRC patients benefited from anti-EGFR agents. First, *KRAS* exon 2 mutations were unveiled to confer resistance to cetuximab and panitumumab, and later *KRAS* exon 3 and 4, *NRAS* exons 2, 3, and 4, and *BRAF* mutations were also established as resistance mutations to EGFR blockade. Subsequently, it also became clear that patients with right-sided mCRC derived less benefit from anti-EGFR agents. More recently, other molecular alterations such as MSI, *HER-2* amplification/overexpression, and *NTRK* fusions, among others, have been shown to be targetable in mCRC. Therefore, it is of utmost importance that clinicians are aware of the molecular biology of CRC and the biological rationale behind treatment decisions in mCRC.

### 2.1. The EGFR-related pathway

EGFR belongs to the erythroblastosis oncogene B (*ErbB*)/human epidermal growth factor receptor (HER) family, which consists of four members: *ErbB1* (EGFR/HER1), *ErbB2* (Neu/HER2), *ErbB3* (HER3), and *ErbB4* (HER4) [7,8]. Overexpression of EGFR has been observed in 25-77% of CRCs and might also associate with poor prognosis [9-11]. The typical *ErbB* receptor consists of 3 domains: a ligand-binding domain outside the cell, a transmembrane domain and an intracellular domain with distinct tyrosine residues in the C-terminal region where subsequent phosphorylation may take place on activation [12].

The union of the EGF ligand with the EGFR initiates the activation of the EGFR and its subsequent phosphorylation (pEGFR), allowing the formation of a coupling site for GRB2 and its union to SOS in the cytosol. The resulting complex (pEGFR united to SOS) promotes nucleotide exchange and *RAS* activation [13-15]. Subsequent RAF activation leads to phosphorylation of mitogen-activated protein kinase (MAPK) and activation of extracellular signal-related kinase (ERK), which might then translocate inside the nucleus to regulate the expression of transcription factors and the activation of specific genes that stimulate cancer progression. Downstream intracellular signaling pathways, including the Phosphatidylinositol-4,5-bisphosphate 3-kinase (PI3K)/AKT and JAK/signal transducer and activator of transcription (STAT)3 pathways, are also triggered to regulate cell growth, survival, and migration [16-18].

### 2.2. RAS status: Cornerstone mutation of mCRC

*RAS* proteins are part of a large family of small guanosine triphosphate (GTP) nucleotide-binding proteins [19]. The human RAS superfamily consists of more than 100 members that can be divided into six subfamilies, the most characteristic being *HRAS*, *NRAS*, and *KRAS* [20,21]. *KRAS* mutations are the most common predictive mutations, occurring in 40-45% of all mCRC [22]. The vast majority of *KRAS* mutations (85-90%) occur in codons 12 and 13 of exon 2, while the rest are found in codons 61, 146, and other residues [23,24]. The patients with *KRAS* mutations are most often adult women with mucinous differentiation [25]. *HRAS* and *NRAS* mutations are found in <5% of patients [26]. Patients with *NRAS* mutations are also usually women with left sided tumors [27].

Among *RAS* family members, *KRAS* is the only one which is essential for normal development, as demonstrated by genetic studies in laboratory animals [13,28,29]. KRAS can be expressed as two different variants: 4A and 4B. Variant 4B is the dominant form, which is commonly known as *KRAS*, a cell membrane-bound GTPase that alternates between an active and an inactive form. GTPase activator proteins hydrolyze the nucleotide GTP leading to phosphate loss and formation of nucleotide guanosine diphosphate (GDP), while guanosine nucleotide exchange factors (GEF) facilitate the exchange of GDP to GTP. Both factors control the transition from the inactive form of *KRAS* to its active form [19,30,31].

Mutations in specific codons in *KRAS* alter the position of a glutamate residue at codon 61 [19,32]. *KRAS* activation occurs without the need for binding of the phosphorylated EGFR protein complex to GEF SOS, resulting in the reduction of the GTPase activity of *KRAS*, decreasing the hydrolysis rate of GTP approximately 3-9 times compared to the non-mutated *KRAS* [21,33,34]. The main effect of *RAS* signaling occurs through the RAF/MEK/ERK pathway and the secondary molecular cascade to the PI3K/AKT pathway, which control growth processes and cell survival [13]. This is achieved in part by activating transcription factors that promote ERK-regulated cell cycle progression and by AKT-mediated inactivation of apoptosis [35].

Various studies have shown that *RAS* mutations play a significant role in cell proliferation, suppression of apoptosis and in changing the tumor microenvironment that ultimately promote tumor cell survival and progression of cancer. Additional functions of *KRAS* have been described, such as regulation of cell migration, endocytosis, cytoskeleton modification, and calcium signaling [36-38].

### 2.3. BRAF mutations: A particular event

*BRAF* mutations are found in 8%–10% of CRCs and do not usually overlap with *RAS* mutations, being considered mutually exclusive [39-41]. Two-thirds of the patients with *BRAF* mutations have primary tumors on the right side of the colon, being associated with a higher frequency of peritoneal involvement, lymph node metastases, and a lower frequency of pulmonary metastases [40]. Up to one-third of *BRAF* mutant tumors harbor a high MSI (MSI-H) and the same proportion of MSI-H tumors have *BRAF* mutations. *BRAF* appears to act through the dentate/methylating pathway and, indeed, *BRAF*-mutant tumors are characterized by the methylation of CpG islands that cause the epigenetic repression of tumor suppressor genes, known as CpG island methylating phenotype tumors [42-44]. The *BRAF* oncogene encodes a serine/threonine kinase that acts in the MAPK pathway. *BRAF* mediates its effect through the activation of MAPK, thus promoting cell proliferation. *BRAF V600E* mutations account for 90% of *BRAF* mutations in CRC. Their occurrence has been associated with older adult women with a history of smoking [45]. The *BRAFV600E* mutation is the result of the transversion of thymidine to adenine at nucleotide 1799 in the kinase domain, resulting in a substitution of valine for glutamate leading to constitutive activation of MEK and uninhibited EGFR-independent cell proliferation [46,47]. The fact that *BRAF* and *KRAS/NRAS* are mutually exclusive mutations in CRC supports the hypothesis that *BRAF* is the main effector of *KRAS/NRAS* in the MAPK pathway and that both have similar effects on tumorigenesis [48,49].

### 2.4. The VEGF/VEFGR pathway

Angiogenesis, a physiological process by which new vessels form or reform from existing vessels, plays a key role in tumor initiation, growth, and metastasis. Angiogenesis is under a complex regulation involving various proangiogenic and antiangiogenic factors, such as VEGF [50-52]. The VEGF family consists of five members (VEGF-A, VEGF-B, VEGF-C, VEGF-D, and PIGF), which may bind to endothelial cells via tyrosine kinase VEGF receptors (VEGFRs). VEGF, VEGFR, VEGF-A, VEGF-B, and PIGF contribute predominantly to angiogenesis, while VEGF-C and VEGF-D tend to regulate lymphangiogenesis. VEGFRs are divided into three types, VEGFR-1, VEGFR-2, and VEGFR-3, along with the non-tyrosine kinase co-receptors neuropilin-1 and NP-2 [53-56].

VEGFR-1 regulates cell differentiation, migration and promotes differentiation of epithelial cells [57,58]. Meanwhile, VEGFR-3 mediates the differentiation, migration, proliferation, and survival of lymphatic endothelial cells [59]. VEGFR-2 is actively involved in vascular formation and is mostly expressed in blood and lymphatic epithelial cells [60]. VEGF-A and VEGF-B mainly bind to VEGFR-1 and VEGFR-2. VEGFR-1, VEGFR-2, and VEGFR-3 activation leads to phosphorylation of tyrosine residues and activation of various pathways, including the *RAS*/RAF/ERK/MAPK pathways that promotes epithelial cell growth, and the PI3K/AKT pathway, by which cell apoptosis may be avoided and contributes to the differentiation, proliferation, migration, and apoptosis resistance of epithelial cells [52,56,59]. The proangiogenic effects of VEGF-VEGFR are important in local sites, favoring tumor progression and migration, as well as for neovascularization in metastatic sites to support cancer survival and growth [61].

## 3. KRAS, NRAS, and BRAF Status: Personalizing the First-line Treatment Today

Fluoropyrimidines are a main part of the backbone of combination regimes in mCRC. Randomized clinical trials have shown that fluoropyrimidine-based combinations with oxaliplatin or irinotecan (FOLFOX, FOLFIRI or XELOX) in the first-line significantly improve treatment efficacy, achieving a response rate of 34-55%, a time to progression of 7-8 months and a median overall survival (mOS) of 14-21 months. The triple therapy with FOLFOXIRI has been compared with FOLFOX or FOLFIRI, demonstrating superiority for FOLFOXIRI in terms of efficacy outcomes, notably with a 25% survival and a 30% increase in response rate. However, because of marked grade 3-4 toxicity, triple therapy is reserved for patients with mCRC with a good performance status, that are highly symptomatic and were the main therapy objective is response rate. In addition, in the past 15 years, monoclonal antibodies have been added to first-line chemotherapy regimens in mCRC [62-64]. Inhibition of the EGFR by panitumumab or cetuximab leads to *KRAS* becoming GDP-bound, which inhibits downstream signaling [39]. Cetuximab was approved by the FDA in mCRC in 2004, although it was not until 2012 that it was approved in the first-line setting. The OPUS and COIN trials demonstrated a higher objective response rate (ORR) with the first-line chemotherapy plus cetuximab in exon 2 *KRAS* wild-type mCRC patients in comparison with chemotherapy. However, no differences were reported in mOS and median progression-free survival (mPFS) [36,65]. The CRYSTAL study demonstrated numerically longer mOS (14.1 vs. 10.3 months, HR=0.91, *P*=0.7), and mPFS (8 vs. 5.6 months, HR=0.93, *P*=0.86), and a higher ORR (19.2% vs. 15.2%, *P*<0.0001) with FOLFIRI/Cetuximab in comparison with FOLFIRI alone in patients with *KRAS* wild-type*/BRAF* mutant. Benefit was superior with cetuximab regimen in patients with *KRAS* wild-type/*BRAF* wild-type mCRC for mOS (25.1 vs. 21.6 months, HR=0.83, *P*=0.0549), mPFS (10.9 vs. 8.8 months, HR=0.68, *P*=0.0016) and ORR (61% vs. 42.6%, *P*<0.0001) [66]. The FIRE-3 trial evaluated the anti-EGFR cetuximab versus the anti-VEGF bevacizumab, both in combination with chemotherapy in patients with *KRAS/NRAS/BRAF* wild-type mCRC. 8 months longer mOS was achieved with cetuximab/FOLFIRI in comparison with bevacizumab/FOLFIRI (33.1 vs. 25 months, HR=0.697, *P*=0.0059). In addition, ORR with cetuximab/FOLFIRI was higher (72% vs. 56.1%, OR 2.01, 0=0.003) [67]. Furthermore, anti-EGFR agents have also demonstrated its value in the neoadjuvant setting. The CELIM trial evaluated FOLFOX6/FOLFIRI and cetuximab in mCRC with unresectable liver metastasis, achieving a response rate of 70% in *KRAS* codon 12/13/61 wild-type patients, allowing liver resection in 93% of the studied population [68]. Likewise, the POCHER trial found a similar response rate (79%) with chemotherapy/cetuximab in a similar population [69]. In mid-2021, the JACCRO trial achieved a median depth of response of 57.4% in *KRAS* wild-type mCRC with mFOLFOXIRI/Cetuximab, compared to a 46% with mFOLFOXIRI/Bevacizumab (*P*=0.001). Median depth of response was higher in the left vs. right-sided mCRC (60.3% vs. 46.1%, *P*=0.0007) [70].

Panitumumab was approved by the FDA for first-line mCRC in 2014. The PRIME trial evaluated panitumumab/FOLFOX4 versus FOLFOX4 in the first-line mCRC. This trial found superior mOS (23.9 vs. 19.7 months, HR=0.83, *P*=0.072), mPFS (9.6 vs. 8 months, HR=0.80, *P*=0.02) and ORR (57% vs. 48%, *P*=0.018) with panitumumab/FOLFOX4 in *KRAS* wild-type mCRC [34]. Further analysis reported modest benefit in mPFS (6.9 vs. 5.5 months, HR=0.68, *P*=0.006) and mOS (18.7 vs. 15.4 months, HR=0.83, *P*=0.15) with panitumumab/FOLFOX4 in patients with *KRAS/NRAS/BRAF* wild-type [71]. The PEAK trial evaluated panitumumab/chemotherapy versus bevacizumab/chemotherapy in *KRAS/NRAS* wild-type mCRC patients. mPFS with panitumumab/chemotherapy was 13.1 months compared to 10.1 months (HR=0.61, *P*=0.0075) with bevacizumab/chemotherapy. mOS was also superior survival benefit (41.3 vs. 28.9 months, HR=0.63, *P*=0.058) [72]. In the neoadjuvant setting, the phase II VOLFI trial reported the highest ORR in mCRC with liver metastasis to date. FOLFOXIRI plus panitumumab achieved an ORR of 85.7% compared to 60.6% with FOLFOXIRI alone (OR=3.9; *P*=0.0098). In addition, ORR achieved 90.6% in left-sided and 86% in *RAS*/*BRAF* wild-type mCRC [73]. Anti-EGFR plus chemotherapy became the treatment of choice in *RAS* and *BRAF* wild-type mCRC [39].

The AVF 2107 phase III study evaluated the combination of bevacizumab/FOLFIRI versus FOLFIRI alone in first-line mCRC. mPFS (10.6 vs. 6.2 months, HR=0.54, *P*<0.0001), mOS (20.3 vs. 15.6 months, HR=0.66, *P*<0.001), and ORR (45% vs. 35%; *P*=0.004) were superior with bevacizumab/FOLFIRI [74]. Likewise, the NO16966 trial reported a longer mOS (21.2 m vs. 19.9 m, HR=0.89, 0.76-1.03, *P*=0.07) and mPFS (9.4 m vs. 8.0 m, HR=0.83, *P*=0.023) with bevacizumab/FOLFOX4 compared to FOLFOX4 alone [75]. In the phase III AVEX trial, bevacizumab remained relatively safe and effective when treating elderly patients with mCRC, achieving a mPFS of 9.1 months with capecitabine/bevacizumab and 5.1 months with capecitabine alone (HR=0.53, *P*<0.0001). Grade 3-4 adverse events were 40% in the combination group and 22% in the capecitabine-alone group [76]. A meta-analysis of 6 randomized trials including 3060 patients concluded that bevacizumab achieved a significantly longer PFS (HR=0.72, *P*<0.00001) and OS (HR=0.84, *P*<0.00001). Further investigation found that both *KRAS* mutant and *KRAS* wild-type mCRC may benefit from bevacizumab. A pooled analysis evaluated the efficacy and safety of bevacizumab in mCRC. This study included 3763 patients from different randomized trials and the addition of bevacizumab to chemotherapy was associated with statistically significant increases in OS (HR=0.80, 0.71-0.90) and PFS (HR=0.57, 0.46-0.71). The effects on OS and PFS across subgroups defined by the chemotherapy backbone, extent of disease, age, ECOG and *KRAS* status were consistent with the overall analysis. Interestingly, the benefit with bevacizumab was found even in mCRC patients with *KRAS* mutation [74,77-79].

In patients with mCRC who responded to bevacizumab plus chemotherapy, maintenance therapy with bevacizumab may be considered. In the prospective BRiTE study in patients with first-line mCRC, maintenance with bevacizumab dramatically improved mOS compared with no maintenance (31.8 vs. 19.9 months, HR=0.48, *P*<0.001) [80]. The MACRO trial reported that continuing with bevacizumab plus capecitabine or bevacizumab alone after bevacizumab plus CAPOX achieved a similar mOS (23 vs. 19 months, HR=1.09, *P*=0.38) [81]. In the phase III CAIRO3 trial, maintenance with bevacizumab plus capecitabine after bevacizumab/CAPOX achieved a mPFS2 of 11.7 months compared to 8.5 months in the capecitabine-alone group (HR=0.63, *P*<0.001) [82]. Maintaining bevacizumab beyond progression has also been evaluated in different trials. The ML18147 study reported that patients who continued bevacizumab plus chemotherapy after progression achieved a modest improvement in mOS compared to chemotherapy alone (11.2 vs. 9.8 months, HR=0.81, *P*=0.0062) [83]. However, in the BEBYP trial, the combination of bevacizumab plus chemotherapy beyond progression in the first-line setting achieved only a modest improvement in mPFS (6.8 vs. 5 months, HR=0.7, *P*=0.01) [84]. Finally, other antiangiogenic agents such as aflibercept and ramucirumab have been tested, respectively, within the VELOUR and RAISE studies in the second-line setting [85,86].

The most relevant studies of first-line therapy in mCRC will be discussed below and are summarized in Table 1.

**Table 1:** Most relevant trials of first-line therapy in biomarker-selected populations

Trial	Phase	Treatment	Target	mPFS	mOS
CRYSTAL	III	FOLFIRI+Cetuximab vs. FOLFIRI	EGFR	10.9 m 8.8 m *P*=0.0549	25.1 m 21.6 m *P*=0.0016
PRIME	III	FOLFOX+Panitumumab vs. FOLFOX	EGFR	6.9 m 5.5 m *P*=0.006	18.7 m 15.4 m
PEAK	II	FOLFOX+Panitumumab vs. FOLFOX+Bevacizumab	EGFR	13 m 10.1 m *P*=0.0075	41.3 m 28.9 m *P*=0.058
FIRE-3	III	FOLFIRI+Cetuximab vs. FOLFIRI+Bevacizumab	EGFR	10 m 10.3 m *P*=0.77	33.1 m 25 m *P*=0.0059
AVF-2107	III	FOLFIRI+Bevacizumab vs. FOLFIRI	VEGFR	10.6 m 6.2 m *P*<0.0001	20.3 m 15.6 m *P*<0.001
NO16966	III	FOLFOX+Bevacizumab Vs. FOLFOX	VEGFR	9.4 m 8.0 m *P*=0.023	21.2 m 19.9 m *P*=0.07
CHECKMATE-142	II	Ipililumab/Nivolumab	PD-1/CTLA-4	NR	NR
KEYNOTE-177	III	Pembrolizumab vs. Chemotherapy	PD-1	54 m 24.9 m *P*<0.002	NR 36.7 m

EGFR: Epidermal growth factor receptor, VEGFR: Vascular endothelial growth factor receptor, PD-1: Programmed cell death protein-1, CTLA-4: Cytotoxic T-lymphocyte antigen 4, NR: Not reached, mPFS: Median progression free survival, mOS: Median overall survival. vs.: Versus

## 4. Sidedness Matters in mCRC: Right Versus Left Colon Cancer

Primary tumor sidedness is a main clinical criterion that, combined with *RAS* and *BRAF* mutational status, is usually considered when choosing the best therapeutic option in mCRC [87]. It is well known that left and right colon cancers have a different embryological origin. Left colon develops from the hindgut, receiving irrigation from the inferior mesenteric artery, while the right colon develops from the midgut and is irrigated from the superior mesenteric artery. Right and left colon cancers have different molecular backgrounds and distinct clinical behaviors. Indeed, compared to left sided CRC, right colon cancer has a higher frequency of the *BRAF, KRAS, PIK3CA* mutations, more commonly harbors MSI-H, and more frequently shows mucinous differentiation. On the other hand, left colon cancer has a higher EREG expression, and more commonly shows18q loss, 20q gain, and EGFR and HER2 gains [88]. This particular molecular profile confers right colon cancer a worse prognosis and response to therapy. Left colon cancer usually has a less aggressive evolution and better prognosis. Furthermore, right colon cancer shows less benefit from anti-EGFR agents even in *RAS* and *BRAF* wild-type tumors [89,90]. Therefore, anti-VEGFR agents are the biologicals of choice to be combined with chemotherapy in right-sided mCRC independently of *RAS* and *BRAF* status and in left-sided mCRC with *RAS* or *BRAF* mutations, while anti-EGFR agents are the treatment of choice in the left-sided *RAS*/*BRAF* wild-type mCRC [91-93].

## 5. The New Weaponry: Immunotherapy in MSI-H CRC

The terminology regarding MSI is not homogeneous. However, MSI is commonly described as a hyper-mutable phenotype, resulting from a defective DNA mismatch repair (MMR) system [94]. The MMR system is responsible for correcting errors in DNA replication. Mutations in MMR genes lead to the accumulation of mutations favoring malignant transformation. Therefore, MSI-H tumors are associated with the production and accumulation of hundreds of somatic mutations, which lead to a high neoantigen exposure that favor the initiation of a robust antitumor immune response [22,23]. Response to immunotherapy has been studied in this particular population in recent years.

**Figure 1:**
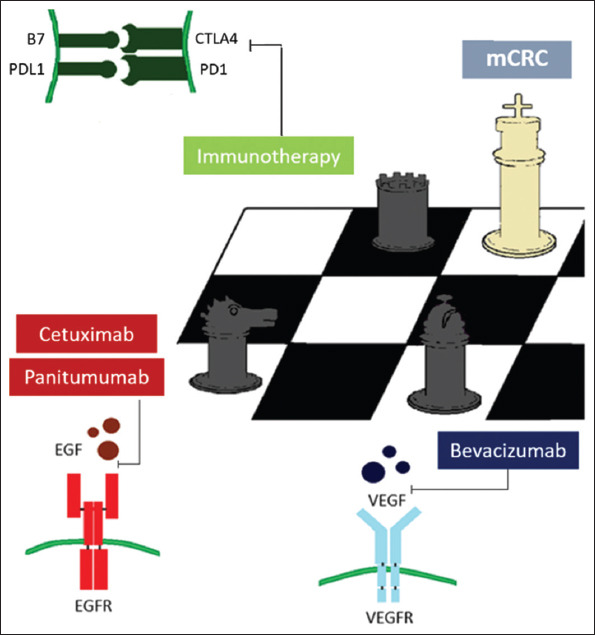
Checkmating the king with the knight and bishop is one of the most complicated chess moves. The first monoclonal antibody approved by the FDA for the treatment of mCRC was bevacizumab in 2004. Subsequently, cetuximab and panitumumab joined the fight, with their corresponding approvals in 2009 and 2014, respectively. The recently FDA-approved pembrolizumab and nivolumab/ipilimumab add to the present weaponry against mCRC. VEGFR: Vascular endothelial growing factor receptor, EGFR: Epidermal growth factor receptor, mCRC: Metastatic colorectal cancer.

As when playing chess, and as depicted in Figure 1, immunotherapy has recently been added to the existing weaponry to combat mCRC.

The Keynote 028 and Keynote 164 trials demonstrated the efficacy of pembrolizumab, an anti-PD1 agent, in patients with heavily pretreated MSI-H mCRC. Furthermore, a whole exome sequencing study within Keynote 028, found that patients with DNA mismatch repair had a much higher mutational load than patients without DNA repair deficiency (1782 vs. 73, *P*=0.007). Checkmate-142, a multi-cohort phase II trial, evaluated the ORR with nivolumab, another anti PD-1, in heavily pretreated patients with MSI-H mCRC. ORR was 31.1% (95% CI 20.8-42.9) while the median duration of response (DOR) was not reached. Treatment with nivolumab achieved a 1-year PFS of 50.4% and a 1-year OS of 73.4%. Another cohort from Checkmate-142 evaluated the addition of ipilimumab, an anti CTLA-4 agent. The combination of ipilimumab/nivolumab in heavily pretreated mCRC achieved an ORR of 55% (95% CI, 45.2-63.8), and a 1-year OS and PFS of 71% and 85%, respectively; and the combination showed a favorable impact in quality of life, with grade 3-4 adverse events occuring in 32% of patients [95-99].

**Figure 2:**
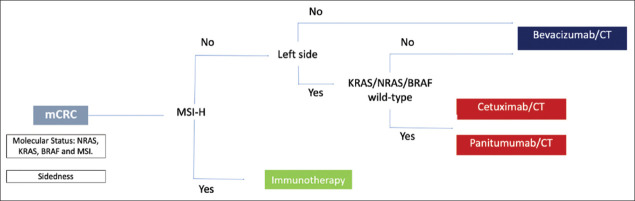
Molecular status and primary tumor sidedness are relevant predictive factors in mCRC. Pembrolizumab and Ipilimumab/nivolumab showed benefit in patients with MSI-H mCRC. Patients with the left sided wild-type *RAS*/*BRAF* mCRC are the most benefited with Cetuximab and Panitumumab (Anti-EGFR agents). If patients harboring any mutation (*NRAS*, *KRAS* or *BRAF*) and/or with a right-sided mCRC, bevacizumab (anti-VEGFR agent) is the best biological companion. CT: Chemotherapy, mCRC: Metastatic colorectal cancer, VEGFR: Vascular endothelial growing factor receptor, EGFR: Epidermal growth factor receptor, MSI-H: Microsatellite instability high.

Another cohort from Checkmate-142 evaluated the combination of nivolumab/ipilimumab in the first line setting. ORR achieved 60% (95% CI, 49-78%) with a non-reached median DOR and a 1-year PFS, and 1-year OS of 77% and 83%, respectively [100]. Updated results after a median follow-up of 29 months, reported a 69% ORR and a 2-year PFS and OS of 74% and 79%, respectively, while median DOR, PFS and OS had not been reached yet. Of note, only 7% of patients developed grade 3-4 adverse events with this regimen, and the nivolumab/ipilimumab combination was finally FDA-approved as first-line therapy of MSI-H mCRC in July 2018 [101]. More recently, the Keynote-177 study evaluated pembrolizumab versus chemotherapy in the first-line MSI-H mCRC. Median PFS was twice longer with pembrolizumab than with chemotherapy (16.5 months vs. 8.2 months, HR=0.6 [95% CI 0.5-0.80], *P*<0.002). ORR was also higher with pembrolizumab (43% vs. 33%, *P*=0.275) and median DOR had not been reached in the immunotherapy arm. Toxicity with pembrolizumab was easily manageable. Notably, at 24-months follow-up, 48% of patients in the pembrolizumab arm remained free of disease progression compared to 19% in the chemotherapy arm [102]. Keynote-177 was considered a practice-changing trial and pembrolizumab was added to the therapeutic options for MSI-H mCRC (Figure 2), being FDA-approved for first-line mCRC in June 2020. In addition, after 36 months of follow-up, the pembrolizumab arm achieved a mPFS of 54 months, compared to 24.9 months in the chemotherapy arm. The 3-year PFS rate reached 60% in the pembrolizumab arm compared to 39% in the chemotherapy arm (HR=0.61, 0.44 – 0.83). ORR was also higher with pembrolizumab (45.1%) compared to chemotherapy (33.1%).

On the other hand, the anti PD-1/anti CTLA-4 combination, as already mentioned, achieved a much higher ORR (69%), although a significant benefit in OS is unclear yet. 3-year OS rate was higher with pembrolizumab compared with the chemotherapy arm, although without statistical significance (61% versus 50%, HR=0.74, 0.53-1.03). mOS was not reached with pembrolizumab, while patients in the chemotherapy arm achieved a 36.7 months mOS [103]. Further follow-up to determine the benefit in OS of the pembrolizumab arm is still needed.

## 6. Potential New Weapons: Future Perspectives in the First-line Treatment

All future potential treatment options are summarized in Table 2.

**Table 2:** Randomized trials with new targeted therapies in mCRC

Target pathway	Trial	Phase	Treatment	Endpoint	Other results
HER2 Overexpression/Amplification	HERACLES-A	II	Trastuzumab/lapatinib	RR=29.6%	mPFS=21 w, mOS=46 w
	HERACLES-B	II	Pertuzumab/TDM-1	RR=9.7%	mPFS 4.1 m
	MyPAthway	IIa	Trastuzumab/Pertuzumab	RR=32%	mPFS=5.3 m, mPFS=14m
	DESTINY-CRC01	II	trastuzumab deruxtecan	RR=45.3%	mPFS=6.9 m, mOS=15.5 m
Targeting PI3K pathway	Rosen *et al*.	I	Apitolisib/CT	RR=3/30 pts	Safety=AEs in>20%
	Yang *et al*.	Ib	Buparlisib/FOLFOX	Safety	-
	Coleman *et al*.	I	Sapanisertib/Metformin	RR=0/2 pts	Safety
Loss of PTEN	Jansen *et al*.	I	Decitabine	Safety	-
	Garrido-Laguna *et al*.	I/II	Decitabine/Panitumumab	Safety	-
Targeting HGF/MET pathway	Van Cutsem *et al*.	I/II	Rilo or Ganitumab/Pani	RR=31%/22%	mPFS=5.2/13.8 m, mOS=5.3/10.6
	Bendell *et al*.	II	Onartuzumab/CT	Safety	m
	Eng *et al*.	I/II	Tivantinib/Cetu/CT	mPFS=8.3 m	mDOR=6.4 m Safety

mCRC: Metastatic colorectal cancer, HER2: Human epidermal growth factor receptor-2, RR: Response rate, mPFS: Median progression free survival, mOS: Median overall survival, mDOR: Median duration of response, CT: Chemotherapy, Rilo: Rilotumumab, PTEN: Phosphatase and tension homolog, HGF: Hepatocyte growth factor, MET: mesenchymal-epithelial transition

### 6.1. Targeting HER2 overexpression/amplification

HER2 overexpression/amplification is found in 1.3-6.3% of patients with CRC, especially those with *RAS* and *BRAF* wild-type left-sided tumors [104-108]. HER2 overexpression is most commonly analyzed by immunohistochemistry (IHC), while its amplification is usually determined by fluorescent *in situ* hybridization (FISH) [109]. Unlike other neoplasms, criteria for positivity have not yet been standardized in CRC. Recently, an international collaborative project established as criteria for HER2 positivity in CRC an IHC score of 3+ or 2+ associated with a FISH HER2/CEP17 ratio ≥2.0 in >10% of tumor cells [110].

The HER2 oncogene is located in the 17q21 chromosome and encodes a transmembrane receptor tyrosine kinase. HER2 is a member of the human epidermoid receptor family that includes the EGFR (HER1), HER2, HER3, and HER4 receptors [111]. HER2 has no known ligand but can form heterodimers with EGFR, HER3, and sometimes HER4. Following dimerization, the intracellular tyrosine residues autophosphorylate and subsequently trigger a cascade of multiple important signaling pathways including *RAS*/RAF/MEK/ERK, PI3K/AKT/mTOR, tyrosine Src kinase, and STAT pathways. Since HER2 overexpression activates almost constitutively, part of the downstream signaling that is shared by EGFR, this explains the resistance to anti-EGFR agents of HER2 positive mCRC [112-115].

Reports indicate that patients with with RAS/BRAF wild-type, HER2 amplified, mCRC have shorter PFS and OS than those without HER2 amplification. Results of anti-HER2 therapies in mCRC have been contradictory [116,117]. The HERACLES-A study found benefit (ORR 29.6%) with dual anti-HER2 blockade with trastuzumab and lapatinib in a cohort of patients with HER2-positive heavily pretreated RAS wild-type mCRC [118]. However, the HERACLES-B study did not find a positive impact on survival (PFS 4.1 months) or response rate (ORR 9.7%) with the combination of pertuzumab and T-DM1 in a similar population [119]. Treatment with trastuzumab and pertuzumab achieved a 32% ORR in patients with HER2-amplified mCRC enrolled within the MyPAthway program [120]. Recently, the DESTINY-CRC01 trial reported benefit with trastuzumab deruxtecan in pretreated patients with HER2 positive (IHC 3+ or IHC 2+/ISH+) mCRC. ORR was 45.3%, mPFS 6.9 months and mOS 15.5 months [121]. Siena *et al*. [122] coherently suggest the possible incorporation of anti-HER2 agents as first-line therapy in a near future, although stronger evidence is still needed. Trastuzumab and new anti-HER agents, such as pyrotinib and zanidatamab, are being currently studied in first-line clinical trials (NCT00003995, NCT03929666, NCT04380012, NCT03043313, NCT03365882).

### 6.2. Targeting PI3K pathway

PI3K is a key component of the PI3K/AKT1/MTOR pathway with an important role in CRC pathogenesis [123,124]. Gain-of-function mutations in *PIK3CA* (PI3K catalytic subunit alpha gene) activate the p110a enzyme, the key catalytic subunit of *PI3K*, stimulating the AKT-MTOR pathway and resulting in cancer growth and proliferation [125]. Mutations in the helicase and kinase domains of exons 9 and 20 of *PIK3CA* occur in 10-20% of CRC and are associated with other molecular alterations such as *BRAF* and *KRAS* mutations and high-grade CpG island methylator phenotype. *PIK3CA* mutation frequency increases from the rectum to the proximal colon and its prognostic value is controversial [126-131]. A number of studies indicate that anti-EGFR agents show no benefit in survival in patients with exon 20 *PIK3CA* mutations regardless of *RAS* and *BRAF* status [132,133].

Some PI3K inhibitors have been developed and evaluated in phase I trials. Copanlisib prevents the growth of malignant cells through the induction of apoptosis via protein p53 upregulated modulator of apoptosis (PUMA) [134]. A phase I study demonstrated a manageable safety profile and a 40% disease control rate (DCR) with copanlisib in a cohort of patients with solid tumors, including mCRC [135]. Dactolisib, a dual PI3K and mTOR inhibitor, binds to the ATP-binding cleft of PI3K and mTOR kinase, inhibiting their catalytic activities [136]. Dactolisib effectively inhibits the growth of human colon cancer cells (SW480) by targeting the PI3K/mTOR signaling pathway and inducing apoptosis [137]. Another phase Ib dose-escalation study evaluated apitolisib, another PI3K inhibitor, in combination with capecitabine (Arm A: 19 patients) or mFOLFOX6 + bevacizumab (Arm B: 11 patients) in advanced solid tumors, including CRC. Partial response was observed in only one mCRC patient with mutations in *PIK3CA* and *KRAS*. Further evaluation in the CRC expansion cohort, found that 2 additional patients achieved partial responses. In general, treatment was well tolerated; the most common grade 3 or higher adverse event was hyperglycemia (40%), followed by stomatitis, hypophosphatemia and neutropenia [138]. Another phase I trial evaluating the safety of PI3K inhibitors in patients with advanced solid tumors, reported that buparlisib in combination with mFOLFOX6 significantly increased toxicity compared to buparlisib or mFOLFOX6 alone, and therefore buparlisib is not being developed further in CRC [139]. However, PI3K inhibitors are still being studied. In mid-2021, a phase I study evaluated sapanisertib in combination with metformin in patients with mTOR/AKT/PI3K pathway alterations and heavily pre-treated advanced solid malignancies. Thirty patients were included (only 2 patients with mCRC) and *PI3KCA* was the most common genomic alteration (27%). Disease control rate was 60% with the combination, although patients with mCRC were not among responders [140]. The complexity of the PI3K/AKT/mTOR signaling network involves numerous feedback loops and extensive crosstalk nodes with other signaling pathways and compensatory pathways, and therefore, unfortunately intrinsic and acquired resistance currently limits the therapeutic efficacy of PI3K inhibitors in mCRC [141]. These agents, alone or in combination, are being studied in the first line setting in ongoing trials (NCT04495621, NCT04753203, NCT02861300, NCT03711058, NCT04753203).

### 6.3. Targeting phosphatase and tension homolog (PTEN) loss

*PTEN* is a multifunctional suppressor protein of the PI3K/AKT pathway [142]. This protein dephosphorylates PI3K products by counteracting the PI3K/AKT signaling cascade. PTEN controls cell proliferation, promotes apoptosis, regulates cell migration/adhesion and the formation of new vasculature [143,144]. Loss of *PTEN* results in the development of cancer due to the activation of the PI3K/AKT pathway and is found in 20-40% of patients with mCRC [145]. *PTEN* alterations seem to be more frequently correlated with right-sided tumors, MSI, *BRAF* mutations, lymph node metastases, and a higher tumor stage [146]. Loss of *PTEN* may be associated with resistance to anti-EGFR treatment, but clinical studies have shown conflicting results [147].

In a recent review, Salvatore *et al*. [146], discuss potential ways of targeting PTEN in CRC. Potentiating PTEN transcription by removing an epigenetic block or modifying the exposure to activating or inhibitory transcription factors is a means of increasing PTEN function [148]. Decitabine, a DNA methyltransferase inhibitor, significantly decreased cell proliferation, induced apoptosis and cell cycle arrest of a colon carcinoma cell line *in vitro* [149]. The safety of decitabine through hepatic arterial infusion was investigated in patients with unresectable liver metastases from solid tumors in a dose escalation phase I clinical trial. Decitabine was administered at 3 different dose levels as a 1-h hepatic arterial infusion in 9 patients (4 with mCRC). Decitabine infusion was safe, with grade 1-2 hematological toxicity being the most frequent treatment-related adverse event with no treatment-limiting adverse events. However, there were no objective tumor responses [150]. DNA methyltransferase inhibitors remove methyl groups from DNA, causing the demethylation of DNA. The combination of decitabine and panitumumab was well tolerated and showed activity in *KRAS* wild-type mCRC patients previously treated with cetuximab in a phase I/II trial [151]. Some of the transcription factors can be pharmacologically stimulated: PPARg (via rosiglitazone), EGR-1 (via irradiation), and NFAT (through butyrate, a fatty acid produced by colonic microbiota fermentation) [152]. At the post-transcriptional level, PTEN expression can be impaired by microRNAs (miRNAs) or RNA-binding protein (RBP). miRNAs bind mRNAs causing loss of PTEN expression and activation of the PI3K/AKT signaling cascade. Modulation of those regulatory RNAs and RNA-RBPs represent a therapeutic strategy aiming at restoring PTEN translation and expression, exploiting its antitumor activity, and increasing cellular drug sensitivity [153]. Some PTEN isoforms originating from different start codon translations have been identified. Of those, PTEN-L was shown to counteract the PI3K/AKT pathway, leading to cell death, both *in vitro* and *in vivo* [154]. Finally, post-translational modifications at specific aminoacidic residues can directly modulate PTEN catalytic or binding activity subsequently impacting on PTEN function [155]. Reverting those post-translational modifications or targeting the enzymes involved could be effective at restoring PTEN function in PTEN positive neoplasms [156]. The long noncoding RNA Linc02023 specifically binds to PTEN and blocks its ubiquitination, promoting CRC cell proliferation and survival. Thus, Linc02023 may serve as a novel therapeutic target for restoring PTEN tumor suppressor activity [157].

### 6.4. Targeting the hepatocyte growth factor (HGF)/mesenchymal-epithelial transition (MET) pathway

The HGF and the tyrosine kinase receptor known as MET factor play an important role in proliferation, survival, metastasis, and acquired resistance to cancer treatment [158]. HGF is produced primarily by mesenchymal tissue and is the only known ligand for MET. Patients with CRC have an elevated serum HGF at diagnosis. MET is a member of the transmembrane surface receptor family expressed on endothelial cells and both normal and malignant epithelial cells [87]. Tissue and serum expression of HGF and elevated levels of MET protein and mRNA associate with a poor prognosis in CRC [159,160]. MET mutations and amplifications represent, respectively, 2-5% and 0.5-2% of all mutations in CRC. Overexpression of HGF/MET mRNA and HGF/MET protein occur in 70% and 50% of CRC tissue samples, respectively. HGF-induced translocation of metastasis-associated in colon cancer 1 from plasma to nucleus and its binding to the MET promoter initiates transcription in the MET pathway [161]. The activation of MET signaling starts with the binding of HGF to the MET receptor at the cell membrane level, triggering the formation of a multifunctional intracellular coupling site from two tyrosine residues that bind to subsequent substrates. Activation of the HGF/MET pathway initiates signaling pathways, including MAPK/ERK, PI3K/AKT, and STAT/JAK, the nuclear factor kB complex, regulates hematopoiesis, and promotes organ regeneration and wound healing [162]. Subsequent studies supported the theory that MET over-activation promotes HGF transcription and expression, leading to subsequent MET activation and expression in a loop manner that can be increased via paracrine HGF produced by reactive stromal cells in the tumor microenvironment or in situations such as hypoxia or inflammation [163].

Unfortunately, clinical trials with HGF and MET inhibitors have shown negative results. A randomized phase I/II trial evaluating panitumumab in combination with rilotumumab, ganitumab, or placebo in patients with *KRAS*-wild type mCRC reported ORRs of 31%, 22%, and 21% respectively, while mPFS and mOS were 5.2 and 13.8 months, 5.3 and 10.6 months, and 3.7 and 11.6 months, respectively. Exploratory biomarker analyzes, including MET and IGF-related protein expression, failed to demonstrate a clear predictive value [164]. A phase II trial of onartuzumab combined with mFOLFOX-6 and bevacizumab did not improve survival in previously untreated MET IHC-positive mCRC and MET expression by IHC was not predictive of response [165]. Another phase I/II trial investigated the addition of the oral MET inhibitor, tivantinib to cetuximab/irinotecan (CETIRI). The combination of tivantinib and CETIRI was well tolerated but did not significantly improve PFS in previously treated *KRAS*-wild type mCRC [166]. Finally, in a phase II study enrolling patients with MET-high-amplified, *KRAS* wild-type mCRC, treated with ≥ 1 prior systemic therapy and showing tumor progression on cetuximab or panitumumab within 3 months before enrollment, treatment with tivantinib plus cetuximab showed only modest results in ORR and PFS [167]. Future trials will evaluate the role of HGF and MET inhibitors in mCRC (NCT03592641, NCT02205398, NCT04515394).

## 7. Conclusions

mCRC harbors molecular alterations that besides being prognostic, also allow physicians to make the most adequate treatment decisions for each patient. *RAS* and *BRAF* mutational status and MSI are at present mandatory determinations in all newly-diagnosed advanced CRCs to choose between anti-EGFR, anti-VEGF, and immune checkpoint inhibitory agents. However, other more recently described alterations such as those in HER2, PIK3, PTEN, and others, have been shown to be targetable and constitute promising therapeutic options in the first-line setting. It is, therefore, of utmost importance that physicians are aware of the rapidly-evolving molecular biology and therapeutic advances in advanced CRC to offer the most appropriate and individualized management approaches for these patients.

### Conflict of Interest

The authors declare no conflicts of interest.
